# Rehabilitation after Rotator Cuff Repair

**DOI:** 10.2174/1874325001711010154

**Published:** 2017-02-28

**Authors:** Ourania Nikolaidou, Stefania Migkou, Christos Karampalis

**Affiliations:** Physical and Rehabilitation Medicine, B' Orthopaedic Department, 424 General Army Hospital, Thessaloniki, Greece

**Keywords:** Rehabilitation, Arthroscopic rotator cuff repair, Shoulder, Scientific rationale, Rehabilitation protocols, Tendon healing

## Abstract

**Background:**

Rotator cuff tears are a very common condition that is often incapacitating. Whether non-surgical or surgical, successful management of rotator cuff disease is dependent on appropriate rehabilitation. If conservative management is insufficient, surgical repair is often indicated. Postsurgical outcomes for patients having had rotator cuff repair can be quite good. A successful outcome is much dependent on surgical technique as it is on rehabilitation. Numerous rehabilitation protocols for the management of rotator cuff disease are based primarily on clinical experience and expert opinion. This article describes the different rehabilitation protocols that aim to protect the repair in the immediate postoperative period, minimize postoperative stiffness and muscle atrophy.

**Methods:**

A review of currently available literature on rehabilitation after arthroscopic rotator cuff tear repair was performed to illustrate the available evidence behind various postoperative treatment modalities.

**Results:**

There were no statistically significant differences between a conservative and an accelerated rehabilitation protocol . Early passive range of motion (ROM) following arthroscopic cuff repair is thought to decrease postoperative stiffness and improve functionality. However, early aggressive rehabilitation may compromise repair integrity.

**Conclusion:**

The currently available literature did not identify any significant differences in functional outcomes and relative risks of re-tears between delayed and early motion in patients undergoing arthroscopic rotator cuff repairs. A gentle rehabilitation protocol with limits in range of motion and exercise times after arthroscopic rotator cuff repair would be better for tendon healing without taking any substantial risks. A close communication between the surgeon, the patient and the physical therapy team is important and should continue throughout the whole recovery process.

## INTRODUCTION

Rotator cuff (RC) pathology is a common cause of shoulder pain and can end up in weakness, shoulder instability and limitation of daily activities (work and sports). Symptomatic disease affects between 4% and 32% of the patients’ population with rotator cuff tears [1]. Although patient’s age, activity level, size of the tear and smoking status influence the decision of management, frequently the preferred method of initial treatment is non-surgical. However, if this management is unsuccessful, surgical repair has been shown to relieve pain and improve function in >90% of patients [2]. Surgical repair of the rotator cuff tear aims to restore the anatomy by repositioning the tendon onto the great tuberosity. Rotator cuff tears’ operational techniques have evolved significantly over the past decades. Open RC repairs were the main choice for most of the 20^th^ century, while later the mini open techniques became the alternative and less invasive gold standard method [3]. Nowadays, the arthroscopic repair is widely spread, least invasive, performed as an outpatient procedure and mainly enables secure fixation and sequentially early return to motion [3, 4]. Arthroscopic technique is now considered to be the standard method although mini-open rotator cuff repair is still performed and has shown equivalent clinical results [5]. Beyond the exact technique used, physical therapy seems to play an important role in the overall final clinical outcome. Postoperative rehabilitation protocols may vary in terms of timing, level of intensity, passive motion or immobilization and time of return to work status [6]. As a result, there are two main proposed approaches regarding the optimal rehabilitation algorithm in the early postoperative phase after rotator cuff surgery. The first is an accelerated program that allows early motion in an effort to reduce postoperative stiffness. The second and most traditional one, suggests more conservative rehabilitation sequence with immobilization for 6 to 8 weeks after surgery in order to protect tendon’s integrity.

## FUNCTIONAL ANATOMY AND BIOMECHANICS

The rotator cuff consists of the tendons of subscapularis, supraspinatus, infraspinatus and teres minor muscles. Its main role is to dynamically stabilize the glenohumeral joint, prevent upward migration of the humeral head, contribute to the initial phase of shoulder abduction and provide internal and external rotation [7, 8].

Understanding the biology and the biomechanical factors of the injured and, of course, of the repaired RC tendon is an essential component, ensuring a successful rehabilitation protocol. Histological studies suggest that there are three phases in RC healing process that occur immediately after surgical repair: inflammatory phase (7 days), proliferative phase (2 to 3 weeks) and maturation or remodeling phase (12 to 26 weeks) [9]. It is important to understand the duration and biology of this healing sequence in order to provide a safe and personally individualized rehabilitation treatment [10]. Following tendon -to- bone fixation, inflammatory cells along with platelets and fibroblasts migrate into the repair site during the first week and proliferate over the next 2-3 weeks. This cellular proliferation and matrix disposition of this phase is regulated by several growth factors [11]. Three to four weeks following repair, the remodeling procedure begins and scar tissue organizes the extracellular matrix turnover [11]. The initial type III collagen formation is slowly replaced by collagen type I, until mature scar tissue is formed [11, 12]. This remodeling process does not reach maximal tensile strength for a minimum of 12-16 weeks post repair. In general, the normal tendon to bone transition is not ever recreated and the repaired RC tendon heals to the greater tuberosity *via* fibrocartilage /scar tissue [13, 14]. Normally, the rotator cuff inserts onto the bone through four distinct transition zones: tendon, unmineralized fibrocartilage, mineralized fibrocartilage and bone. After repair, the tendon heals on to the bone with an interposed layer of fibrovascular scar tissue. The mechanical properties of this fibrous tissue are weaker than the native insertion site and may render repairs prone to failure [15].

## POSTOPERATIVE TREATMENT MODALITIES

The primary goals of postsurgical rehabilitation after RC repair are protection of the healing process and parallel prevention of joint stiffness and muscle atrophy. However, there are not many high-level evidence-based studies in the English literature regarding the postoperative rehabilitation protocols. Some authors support the traditional-conservative program, while others present that both protective and accelerated rehabilitation protocols, based on individual patient, minimize the risk for developing postoperative stiffness without compromising the final result. Ghodadra *et al*. [16] showed that there are many factors that influence rehabilitation, such as surgical approach, quality of the tendon, localization and tear configuration and finally etiology of the rupture (acute *vs* degenerative).

## IMMOBILIZATION

The most common complication after RC surgical repair is stiffness, regardless of an arthroscopic or open technique. Postoperative stiffness can be an undesired outcome, resulting from prolonged immobilization. In general, rehabilitation protocols need to take into account that tendon to bone healing is slow. However, animal models have shown that postoperative rehabilitation reduces the tension of rotator cuff repairs and improves the healing process. There are many discussions about the duration of immobilization. Most authors agree that a period of immobilization 4 to 6 weeks is beneficial during the early postoperative phase [10]. After this initial 6-week period, most protocols suggest the beginning of a more aggressive passive and active range of motion with a proper progression of activity. Some authors suggest early passive motion in order to avoid stiffness, regardless the slow process of tendon attachment to the bone. They suggest that early motion does not negatively affect tendon healing. However, a recent study shows that longer immobilization periods may not increase the incidence of postoperative stiffness [17]. Regarding immobilization and its effect on the range of motion, many authors reported that postoperative stiffness with delayed motion is not a concern and that these patients will eventually regain full range of motion during the rehabilitation phase [18]. On the other hand, several authors suggest earlier and more aggressive rehabilitation protocols, gradually increasing the range of motion. In these protocols, passive range of motion in the limits of pain tolerance, is recommended, under the supervision or direction of a physical therapist, whileslow protocols generally limit early motion by allowing only limited passive range of motion and do not encourage either active participation or the use of CPM device [19].

Another factor that is usually under discussion is the arm position during immobilization. Arm sling in abduction is now widely suggested while many studies have shown that vascularization is improved and tension of the repaired tendon is minimized in this position [20]. Hatakeyama *et al*. showed there is less tension on the superior rotator cuff in 30^ο^ and 45^ο^ compared to 0 and 15^ο^of abduction [21].

## CONTINUOUS PASSIVE MOTION (CPM)

The benefits of continuous passive motion after rotator cuff repair are not clear. Researchers suggest that CPM minimizes postoperative pain and appears to be more significant when it is used in conjunction with traditional physical therapy. Many authors reported no significant difference in clinical outcome of patients treated with CPM during the first 3-4 weeks following rotator cuff repair in comparison to those treated without it. Lastayo *et al*. [22] in a level I randomized controlled study compared the results of CPM in passive range of motion and found no significant differences between the two groups concerning pain and motion disability or isometric strength. In conclusion, CPM may be used to gain motion while minimizing muscle activity, however its long-term benefit is still unclear [23].

## ICE OR CRYOTHERAPY

The role of cryotherapy in the early postoperative period is to decrease pain, swelling, minimize the inflammatory response and improve muscle spasm. Several studies have shown that cryotherapy decreased pain and narcotic medication requirement in the immediate post-operative 24 hours compared to the control group [24].The reason for this is that continuous cryotherapy causes a significant reduction of both glenohumeral joint and subacromial space temperatures at variable times during the first 23 postoperative hours. The effects of cryotherapy seem to be part of the basic analgesic mechanism due to the temperature’s reduction. In conclusion, recent studies endorse the use of cryotherapy at home for 10-14 days after surgery, although there are no well-controlled studies of its continuous application [24].

## AQUATIC REHABILITATION

The benefits and the clinical advantages of water have been utilized for centuries as a mean for rehabilitation, relaxation and training [25]. Aquatic exercises are necessary during the early postoperative phase in maximizing the range of motion in a protective environment. The reason for aquatic exercises being more protective is due to the support of buoyancy and the water’s viscosity [26]. Buoyancy may be used in rehabilitation as assistance, as support and as a resistance. Assisted exercises occur when movements are toward the surface of the water. These exercises are commonly used in increasing mobility, such as when allowing the arm to passively abduct toward the face. For that reasons, the patient may find out that movement patterns occur earlier in the pool than in a gravity environment. Regarding water temperature, recent studies have reported that a warm water pool may improve soft tissue extensibility, by relaxing the muscles in a warm environment. In addition, warm water’s temperature has been found to improve collagen properties [27].

By utilizing a pool for rehabilitation, the patient’s goal of improving range of motion can be achieved easier and much earlier than land-based exercises without compromising the repair integrity [28]. Golland found that exercises performed at 90^ο^ abduction in the water presented less electrographic activity than land-based exercises [29]. Moreover, it is important to note that exercise in the water can still become resistive from assistive if it is performed quickly enough to encounter water’s viscosity resistance. Taking into account the properties of the water, as well as the physiological responses to immersion both at rest and during exercise, the physical therapist can effectively design a rehabilitation program for the patient.

## APPROACH TO DIFFERENT PHASES OF ROTATOR CUFF REHABILITATION

As mentioned, the ultimate goal of a rehabilitation program after rotator cuff repair is to maintain secure the tendon to bone attachment and prevent shoulder stiffness. An ideal rehabilitation program should be based on close communication between the surgeon and the physical therapy team. It is important to know all the necessary details about the surgery, such as the size of the tear, the exact tendons involved, the quality of tissue and the repair technique used, in order to establish a suitable protocol for the patient. A physical therapist should take into account that every patient and every rotator cuff tear is not the same. For example, geriatric patients or large to massive rotator cuff tears may require a slower form of rehabilitation in order to avoid the risk of re-tear. On the other hand, younger patients or smaller tears with good tissue quality are more likely to undergo an accelerated “return”. In addition, there are many risk factors for postoperative stiffness such as adhesive capsulitis, concomitant labral repair, single tendon rotator cuff repair, coexisting calcific tendinitis and diabetes. Patients with all these factors should start an earlier rehabilitation protocol to avoid postoperative stiffness [30]. For these reason, this close communication with the surgeon should be continued throughout the rehabilitation process in order to end up to a successful outcome for the patient.

Lastly, the physical therapist should take into consideration the clinical goals, the pre-operative activities and the needs of each patient. This entails having a lengthy discussion with the patient before and after the surgery in order to cover every aspect of patient’s recovery, to ensure his compliance with the procedure and finally his return to daily activities.

One of the purposes of this review is to present an approach of common rehabilitation protocols after rotator cuff repair. In general, as mentioned above two different post-operative approaches exist: a moderate or accelerated rehabilitation program and a more conservative one. The moderate protocol will be presented in details (phases and exercises). The conservative-traditional protocol is characterized by a delay in each phase of moderate program (2 to 4 weeks) and a restriction of passive range of motion (2-4 weeks).

## MODERATE OR ACCELERATED PROTOCOL

Rotator cuff repair is followed by a program consisted of four commonly used and accepted phases, beginning with the aim to protect mainly the repair in the immediate postoperative phase and gradually return to preoperative activity level [31].

### PHASE I (0 to 4-6 Weeks)

At this phase, one of the main goals is to educate the patient and to establish a close communication in order to understand its personal desires and goals and collaborate throughout the whole recovery process. It is also important, as previously mentioned, to accumulate a detailed medical pre- and intra-operative medical history. Regardless of the determined protocol, at this initial phase, the therapist should focus on maintaining the integrity of the repair.

At phase I, patients are expected to wear their arm immobilizer with the abduction pillow for 6 weeks post-operatively unless specifically directed to do otherwise by the operating surgeon [32]. This depends on whether the tendon was repaired by open, mini-open or arthroscopic technique. This position may enhance regional blood flow by preventing the “wringing out” effect in blood vessels to the tendon and may reduce the distance between the origin and the insertion of the muscle tendon unit so that passive tension on the repair site will be decreased [33]. Moreover, it is prudent during this phase to introduce both active and passive motion of the elbow, wrist, hand and cervical spine.

It is widely described that early passive motion may be beneficial in helping the process of tendon to bone healing. Passive ROM exercises should be performed with gentle oscillations. Pendulum exercises can also be performed by leaning the body with the support of a chair and having the arm dangle in front of the body and by performing clockwise and counter clockwise motion. At this phase, cryotherapy can be helpful regarding postoperative pain and inflammation, so the patients should be instructed on the use of ice at home. Considering the sleeping position, during the first few weeks postoperatively it is usually more comfortable for the patient to sleep in a recliner with a pillow underneath the operative extremity while wearing the abduction sling. Aqua therapy may be also beneficial in the second to sixth week. Furthermore it is equally important the proper education of the patient concerning what to avoid at home. There are many precautions at this phase such as lifting heavy objects, pushing and pulling, excessive shoulder extension, excessive movements behind the trunk stretching or sudden movements, leaning on the elbow supporting of body weight by hand with transfers in and out of bed and chair [34].

The criteria to progress from phase I to phase II is a pain free passive motion. More specific: 1. Passive forward flexion to at least 110^o^ - 125^o^. 2. Passive external and internal rotation in arm immobilizer to at least 25^o^ -45^o^. 3. Passive glenohumeral abduction in arm immobilizer to at least 90^o^.

### PHASE II (4-6 to 10-12 Weeks)

By six weeks postoperatively the inflammatory and repair phases have progressed to the collagen remodeling stage. During this time tendon healing to bone is progressively increasing and the application of low level muscle forces aids in orienting the fibers within the collagen matrix and enhancing the tensile strength of the tendon repair [35]. This phase typically begins 4-8 weeks after surgery, but can be prolonged depending on factors such as quality and size of repair, age of the patient and other comorbidities. The main goals at phase II are continuing passive ROM, introducing active-assisted ROM, improving neuromuscular control and strength, while at the same time minimizing pain and inflammation. At this point, active -assisted ROM exercises, the use of pulleys, canes and self-assisted ROM are widely suggested. For example, supine glenohumeral external and internal rotation with the aid of a cane or supine flexion with the assistance of the uninvolved limb are generally suggested. In addition, alternative assisted active ROM are also initiated. For example, having the patient performing circles on a physioball placed on a table as the patient stands facing the ball with his hand and forearm resting on the ball. Many authors suggest incorporating most cardinal plane motions in this exercise.

Five to 7 weeks after surgery,open-chain proprioceptive exercises can be added to rehabilitation protocol. These exercises help to restore muscle strength and proprioception and are performed with the patient in supine position and the involved upper extremity held in 90^o^ of forward elevation. Then the patient is instructed to draw circles or the alphabet in the air utilizing small, controlled, motions. Moreover in this time, sub-maximal isometric external and internal rotation can be initiated. These exercises are performed holding the arm below shoulder height, elbow flexed to approximately 90^o^, and held in a neutral rotation position. A tower roll is placed between the elbow and trunk and the patient is asked to push into internal or external rotation, resisting with the uninvolved limp, starting at approximately 25% of maximal effort and gradually increasing to 50-70% of maximal effort without pain [36].

During this phase aquatherapy can be more advanced adding active motion as it is considered to be active-assisted ROM in a gravity reduced environment. Studies have shown that shoulder elevation in the water is less active for the rotator cuff compared to dry land exercise [37].

Although the progress, patient in this phase should continue avoiding resistance or strength activities. The criteria to progress to phase III are full active ROM compared to the contralateral arm and no signs of scapular-thoracic dyskinesia [38].

### PHASE III (10-12 to 16-18 Weeks)

Phase III can be called a strengthening phase, taking place approximately 10 to 12 weeks postoperatively. In this phase, the histological remodeling phase is complete and tendon to bone healing is strong enough to allow a strengthening program. The main goals of this phase is having a full passive ROM without pain, optimizing neuromuscular control and improving endurance. The patient must take into consideration that attempts to strengthen a stiff shoulder can cause pain and stress on the repair, so a strengthening program is permitted only when shoulder mobility and ROM are maximized.

During this phase, the patient begins with stretching and strengthening exercises and continues with elastic resistance activities in order to build muscles endurance. These exercises are external rotation, internal rotation, forward flexion and rowing motion, which are performed with a towel roll placed between the arm and trunk. Free exercises can be used to strengthen the biceps and triceps muscles. Generally, patients should have regained at least 80-90% of their range of motion at this phase, unless they had a large or a massive tear requiring lateral mobilization of the tendon or a more extensive procedure. When the patient is pain-free with activities of daily living and tolerates all strengthening exercises without pain, then can continue to phase IV.

### PHASE IV (16-26 Weeks)

Phase IV is the advanced strengthening phase that typically can be initiated approximately 16-22 weeks after rotator cuff repair. At this point the remodeling phase should be complete and the repaired rotator cuff tissue is relatively mature enough to withstand greater forces. Progressive strengthening of repaired rotator cuff can be achieved in many ways. To ensure high levels of infraspinatus and teres minor strength, perform external rotation of the shoulder at 45^o^ of abduction utilizing elastic resistance. Then perform external rotation exercises at 90^o^ of abduction in order to activate supraspinatus muscle. Additionally, the push up with a plus progression from a wall to a chair and then finally to the floor is a more advanced exercise that strengthens the serratus anterior muscle. Finally, upper limb plyometric exercises, known as “jump training” or “plyos”, which for example include a patient throwing and catching a weighted ball against a wall, starting at shoulder height and progressing gradually to overhead. It is thought that these exercises improve neuromuscular control, strength, and proprioception.

At this phase it is also necessary to choose specific exercises that target the scapular stabilizers. An exercise that aims to strengthen the serratus anterior muscle is having the patient standing and facing away from the elastic resistance attachment with the hands held at shoulder width and chest height, holding onto the resistance band. Then the upper extremities are extended forward away from the body at 120 degrees of forward elevation. An additional exercise that strengthens the serratus anterior muscle is the push-up with a plus progression, first beginning with pressing against a wall, then progressing to the edge of a table and finally to the floor

However the restrictions in this phase are overhead lifting and return to competitive sport. Finally, the criteria to return a patient to pro-tear conditions or to normal everyday activities are: 1. Symmetrical ROM and strength, 2. Normalized scapulothoracid kinematics, 3. No pain at rest or with activities.

The postoperative rehabilitation protocols regarding the duration of immobilization, passive/active motion, and aggressive post-operative treatment are still under debate. Huang *et al*. [39] in a recent meta-analysis reported the effects of a post-operative aggressive protocol *versus* those of a traditional rehabilitation protocol. The authors included 6 studies and investigated the effects and the differences of the early passive ROM exercise. The results showed that the aggressive rehabilitation protocol was superior to the traditional protocol regarding the outcomes of overall ROM at 6 months and 1 year after repair, and finally led to greater improvement in shoulder function. However despite the better and earlier functional results, an aggressive rehabilitation protocol may entail higher risks of rotator cuff un-healing and a higher retear rate than the traditional protocol.

The current literature on rehabilitation following rotator cuff repair and its postoperative effects can be summarized in the Table **1**.

## SUMMARY

Rehabilitation following rotator cuff repair begins with a close communication between the surgeon, the patient and the physical therapy team. It is important that this communication should continue throughout the whole recovery process. The physical therapist should collect all the information and details that are useful in order to create an appropriate and successful rehabilitation protocol. These protocols vary between providers with respect to timing of progressing and appropriate therapeutic exercise. Either a conservative or a moderate rehabilitation protocol is selected, the main goals should always be the maintaining of the repair, the minimizing of tendon stress and pain and the faster return to the previous activities of patients life. A conservative protocol is characterized by either a delay in the initiation or/ and restriction of passive ROM while a moderate /accelerated rehabilitation program is characterized by initiation passive ROM on postoperative time. Nowadays all protocols are commonly based on clinical experience and expert opinion rather than scientific rationale.

## Figures and Tables

**Table 1:** Results of different rehabilitation protocols after rotator cuff surgery.

**Study**	**Subjects number**	**Degree and Tear size**	**Surgery type**	**Treatment**	**Duration & frequency**	**Rom elevation** **(early/late)**	**Re-tear rate (early/late)**	**Outcome**
Garofalo *et al*. 2010 [40]	100 (average age = 60)	100% Partialthickness tear (C2-3)	Arthroscopic (no detailed technique)	**Group A:** Control group protocol combined continuous passive motion (CPM) **Group B:** Pendulum movements and progressive passive ABD, ER, flexion	**Both groups** 0 to 4 weeks: Different protocols 5 to 28 weeks: Same protocols	158.1°/151.7° 12 mo: 165.2°/158°	not applicable	CPM reduces joint pain and improves ROM at short-term follow-up
Lee *et al*. 2012 [18]	64 (average age = 55)	100% Full-thickness tear (medium to large)	Arthroscopic Single row	**Group A:** Aggressive early passive ROM (flexion >90° and ER to 30° before week 3) **Group B:** Minimum passive ROM (flexion<90° until week 3)	**Both groups** 0~6 weeks: different passive ROM protocol 6 weeks~: start active ROM	mo: group A, 157.3°; group B, 151.9°. 12 mo: group A, 155.3°; group B, 153°	Group A, 23.3%. GroupB 8.8%.	Limited self-directed rehabilitation best. More aggressive rehabilitation increases the re-tear rate.
Düzgün *et al*. 2011 [41]	29 (average age = 56)	100% Partial-thickness tear Phase 2~3	Side-to-side	**Group A:** Early active movement combinedpreoperative rehabilitation **Group B:** Classical rehabilitation	**Group A:** 8 weeks protocol (4 weeks preoperative treatment) **Group B:** 22 weeks protocol	Not applicable	not applicable	Accelerated protocol led to less pain and more rapid recovery of func- tional level
Kim *et al*. 2012 [42]	95 (average age = 60)	100% Full-thickness tear (small to medium)	Arthroscopic Single row Double row Suture bridge	**Group A:** Controlled early passive motion 1 day after surgery **Group B:** No passive ROM until brace removal (4~5 weeks)	**Group A:** From day 1 **Group B:** From 4 weeks	6 mo: 150.57°/147.14°. 12 mo: 159.75°/ 153.67°	Group A 12% GroupB18%	Beginning early passive ROM <4 wk postoperatively had no effect
Cuff *et al*. 2012 [43]	68 (average age = 63)	100% Full-thickness tear Crescent type	Arthroscopic Suture bridge	**Group A:** Early passive motion 2 days after surgery (Start: flexion<120° and ER<30°) **Group B:** Passive forward flexion and ER 6 weeks after surgery	**Group A:** 3 times/week of PT+ 3 times/day for pendulum **Group B:** 3 times/week PT after 6 weeks+3 times/day for pendulum	6 mo: 158.1°/151.7° 12 mo: 165.2°/158°	Group A 15% GroupB9%	Slight improvement in early ROM but very similar outcomes at 1 y. Slightly better healing observed in the de- layed ROM group

**Abbreviations:** ABD: Abduction; IR/ER: Internal/External Rotation; ROM: Range of Motion; PT: Physical Therapy.

## LIST OF ABBREVIATIONS

CPM: = Continuous Passive Motion
RC: = Rotator Cuff
ROM: = Range of Motion

## References

[1] Boykin R.E., Heuer H.J., Vaishnav S., Millett P.J. (2010). Rotator cuff Disease :Basics of diagnosis and treatment.. Rheumatology Reports.

[2] Lapner P.L., Sabri E., Rakhra K., McRae S., Leiter J., Bell K., Macdonald P. (2012). A multicenter randomized controlled trial comparing single-row with double-row fixation in arthroscopic rotator cuff repair.. J. Bone Joint Surg. Am..

[3] Yamaguchi K., Levine W.N., Marra G., Galatz L.M., Klepps S., Flatow E.L. (2003). Transitioning to arthroscopic rotator cuff repair: the pros and cons.. Instr. Course Lect..

[4] Norberg F.B., Field L.D., Savoie F.H. (2000). Repair of the rotator cuff. Mini-open and arthroscopic repairs.. Clin. Sports Med..

[5] Morse K., Davis A.D., Afra R., Kaye E.K., Schepsis A., Voloshin I. (2008). Arthroscopic *versus* mini-open rotator cuff repair: a comprehensive review and meta-analysis.. Am. J. Sports Med..

[6] Noyes F.R., DeMaio M. (1991). Mangine, R.E. Evaluation-Based protocols: a new approach to rehabilitation.. Orthopedic.

[7] Bateman J.E. (1971). The shoulder and Neck..

[8] Dugas J.R., Campbell D.A., Warren R.F., Robie B.H., Millett P.J. (2002). Anatomy and dimensions of rotator cuff insertions.. J. Shoulder Elbow Surg..

[9] Gulotta L.V., Rodeo S.A. (2009). Growth factors for rotator cuff repair.. Clin Sports Med.

[10] Millett P.J., Wilcox R.B., OHolleran J.D., Warner J.J. (2006). Rehabilitation of the rotator cuff: an evaluation-based approach.. J. Am. Acad. Orthop. Surg..

[11] Carpenter J.E., Thomopoulos S., Alanagan C.L. (1998). Rotator cuff defect healing:a biomechanical and histologic analysis in an animal model.. J Shoulder Elbow Surg.

[12] Lewis C.W., Schlegel T.F., Hawkins R.J., James S.P., Turner A.S. (2001). The effect of immobilization on rotator cuff healing using modified Mason-Allen stitches: a biomechanical study in sheep.. Biomed. Sci. Instrum..

[13] Gohen D.B., Kawamura S., Ehteshami J.R. (2006). Indomethacin and celecoxib impair rotator cuff tendon to bone healing.. Am. J. Sports Med.

[14] Galatz I.M., Sandell L.J., Rothermich S.Y. (2006). Characteristics of the rat supraspinatus tendon during tendon to bone healing after acute injury.. J. Orthop. Res..

[15] Rodepo K.D. (2008). SA 920080.Biological augmentation of rotator cuff tendon repair.. Clin. Orthop. Relat. Res..

[16] Ghodadra N.S., Provencher M.T., Verma N.N., Wilk K.E., Romeo A.A. (2009). Open, mini-open, and all-arthroscopic rotator cuff repair surgery: indications and implications for rehabilitation.. J. Orthop. Sports Phys. Ther..

[17] Parsons B.O., Gruson K.I., Chen D.D (2010). Does slower rehabilitation after arthroscopic rotator cuff repair lead to long term stiffness?. J. Shoulder Elbow Surg..

[18] Lee BG, Cho NS, Rhee YG (2012). Effect of two rehabilitation protocols on range of motion and healing rates after arthroscopic rotator cuff repairs: Aggressive *versus* limited early passive exercises.. Arthroscopy.

[19] Keener JD, Galatz LM, Yamaguchi K (2013). Rehabilitation following arthroscopic rotator cuff repair:A prospective, randomized trial.

[20] Rathbun J.B., Macnab I. (1970). Themicrovascular pattern of the rotator cuff.. J. Bone Joint Surg. Br..

[21] Hatakeyama Y., Itoi E, Pradhan RL, Uragama M., Sato K. (2001). Effect of arm elevation and rotation on the strain in the repaired rotator cuff tendon. A cadavenicstudy.. Am. J. Sports Med..

[22] Lastayo P.C., Wright T., Jaffe R., Hartzel J. (1998). Continuous passive motion after repair of the rotator cuff. A prospective outcome study.. J. Bone Joint Surg. Am..

[23] Raab M.G., Rzeszutko D.O., Connor W., Greatting M.D. (1996). Early results of continuoys passive motion after rotator cuff repair lead to long-term stiffness*?*. J. Shoulder Elbow Surg..

[24] Speer K.P., Warren R.F, Horowitz L. (1996). The efficacy of cryotherapy in the postoperative shoulder.. J. Shoulder Elbow Surg..

[25] (1983). AT S. Duffield’s Exercise in Water..

[26] Thein JM (1998). Aquatic-based rehabilitation and training for the elite athlete.. J. Orthop. Sports Phys. Ther..

[27] Thein J.M., Brody L.T. (2000). Aquatic-based rehabilitation and training for the shoulder.. J. Athl. Train..

[28] Malone TR GW, Zachazewski JE (1996). Muscle: deformation, injury, repair..

[29] Golland A (1981). Basic hydrotherapy.. Physiotherapy.

[30] Fujisawa H., Suenaga N., Minami A. (1998). Electromyographic study during isometric exercise of the shoulder in head-out water immersion.. J. Shoulder Elbow Surg..

[31] Huberty DP, Schoolfield JD, Brady PC (2009). Incidence and treatment of postoperative stiffness following arthroscopic rotator cuff repair.. Arthroscopy.

[32] Hersche O., Gerber C. (1998). Passive tension in the supraspinatus musculotendinous unit after long-standing rupture of its tendon: a preliminary report.. J. Shoulder Elbow Surg..

[33] Millett P.J., Wilcox R.B., OHolleran J.D., Warner J.J. (2006). Rehabilitation of the rotator cuff: an evaluation-based approach.. J. Am. Acad. Orthop. Surg..

[34] Austin V, Zhou H, Guillaumed FS (2013). Physical Therapy and rehabilitation after rotator cuff repair: A review of Current Concepts.. Int. J. Phys. Med. Rehabil..

[35] Long J.L., Ruberte Thiele R.A., Skendzel J.G., Jeon J., Hughes R.E., Miller B.S., Carpenter J.E. (2010). Activation of the shoulder musculature during pendulum exercises and light activities.. J. Orthop. Sports Phys. Ther..

[36] Escamilla R.F., Yamashiro K., Paulos L., Andrews J.R. (2009). Shoulder muscle activity and function in common shoulder rehabilitation exercises.. Sports Med..

[37] Carpenter J.E., Thomopoulos S., Flanagan C.L., DeBano C.M., Soslowsky L.J. (1998). Rotator cuff defect healing: a biomechanical and histologic analysis in an animal model.. J. Shoulder Elbow Surg..

[38] Kelly B.T., Roskin L.A., Kirkendall D.T., Speer K.P. (2000). Shoulder muscle activation during aquatic and dry land exercises in nonimpaired subjects.. J. Orthop. Sports Phys. Ther..

[39] Huang T.S., Wang S.F., Lin J.J. (2013). Comparison of aggressive and traditional postoperative rehabilitation protocol after rotator cuff repair: A meta-analysis.. J. Nov. Physiother..

[40] Garofalo R., Conti M., Notarnicola A., Maradei L., Giardella A., Castagna A. (2010). Effects of one-month continuous passive motion after arthroscopic rotator cuff repair: results at 1-year follow-up of a prospective randomized study.. Musculoskelet. Surg..

[41] Düzgün I., Baltacı G., Atay O.A. (2011). Comparison of slow and accelerated rehabilitation protocol after arthroscopic rotator cuff repair: pain and functional activity.. Acta Orthop. Traumatol. Turc..

[42] Kim D.H., Elattrache N.S., Tibone J.E., Jun B.J., DeLaMora S.N., Kvitne R.S., Lee T.Q. (2006). Biomechanical comparison of a single-row versus double-row suture anchor technique for rotator cuff repair.. Am. J. Sports Med..

[43] Cuff D.J., Pupello D.R. (2012). Prospective randomized study of arthroscopic rotator cuff repair using an early versus delayed postoperative physical therapy protocol.. J. Shoulder Elbow Surg..

